# Low-dose IL-34 has no effect on osteoclastogenesis but promotes osteogenesis of hBMSCs partly via activation of the PI3K/AKT and ERK signaling pathways

**DOI:** 10.1186/s13287-021-02263-3

**Published:** 2021-05-04

**Authors:** Jianxiang Xu, Lifeng Fu, Jinwu Bai, Huiming Zhong, Zhihui Kuang, Chengwei Zhou, Bin Hu, Licheng Ni, Li Ying, Erman Chen, Wei Zhang, Jiaqi Wu, Deting Xue, Weixu Li, Zhijun Pan

**Affiliations:** 1 Department of Orthopedics Surgery, the Second Affiliated Hospital, Zhejiang University School of Medicine, No. 88, Jiefang Road, Hangzhou, 310009 China; 2 Orthopedics Research Institute of Zhejiang University, No. 88, Jiefang Road, Hangzhou, 310009 China; 3 Key Laboratory of Motor System Disease Research and Precision Therapy of Zhejiang Province, No. 88, Jiefang Road, Hangzhou, 310009 China

**Keywords:** Low-dose IL-34, hBMSCs, mBMMs, Osteoblastogenesis, Osteoclastogenesis

## Abstract

**Background:**

Inflammatory microenvironment is significant to the differentiation and function of mesenchymal stem cells (MSCs). It evidentially influences the osteoblastogenesis of MSCs. IL-34, a newly discovered cytokine, playing a key role in metabolism. However, the research on its functional role in the osteogenesis of MSCs was rarely reported. Here, we described the regulatory effects of low-dose IL-34 on both osteoblastogenesis and osteoclastogenesis.

**Methods:**

We performed the osteogenic effects of hBMSCs by exogenous and overexpressed IL-34 in vitro, so were the osteoclastogenesis effects of mBMMs by extracellular IL-34. CCK-8 was used to assess the effect of IL-34 on the viability of hBMSCs and mBMMs. ALP, ARS, and TRAP staining was used to evaluate ALP activity, mineral deposition, and osteoclastogenesis, respectively. qRT-PCR and Western blotting analysis were performed to detect the expression of target genes and proteins. ELISA was used to evaluate the concentrations of IL-34. In vivo, a rat tibial osteotomy model and an OVX model were established. Radiographic analysis and histological evaluation were performed to confirm the therapeutic effects of IL-34 in fracture healing and osteoporosis. Statistical differences were evaluated by two-tailed Student’s *t* test, one-way ANOVA with Bonferroni’s post hoc test, and two-way ANOVA with Bonferroni multiple comparisons post hoc test in the comparison of 2 groups, more than 2 groups, and different time points of treated groups, respectively.

**Results:**

Promoted osteoblastogenesis of hBMSCs was observed after treated by exogenous or overexpressed IL-34 in vitro, confirmed by increased mineral deposits and ALP activity. Furthermore, exogenous or overexpressed IL-34 enhanced the expression of p-AKT and p-ERK. The specific AKT and ERK signaling pathway inhibitors suppressed the enhancement of osteoblastogenesis induced by IL-34. In a rat tibial osteotomy model, imaging and histological analyses testified the local injection of exogenous IL-34 improved bone healing. However, the additional IL-34 has no influence on both osteoclastogenesis of mBMMs in vitro and osteoporosis of OVX model of rat in vivo.

**Conclusions:**

Collectively, our study demonstrate that low-dose IL-34 regulates osteogenesis of hBMSCs partly via the PIK/AKT and ERK signaling pathway and enhances fracture healing, with neither promoting nor preventing osteoclastogenesis in vitro and osteoporosis in vivo.

## Introduction

Bone defects or fracture nonunion, which still has no efficacious way for treatment, is one of the most intractable clinical diseases for orthopedic surgeons [1, 2]. Severe fracture, bone tumor ablation, debridement of a wide range of bone infections, and congenital defects can lead to the failure of fracture healing [3]. Fracture healing is known as an intricate physiologic process that the vascularity at fracture site, mechanical environment, growth factors, scaffolds, and mesenchymal stem cells (MSCs) coordinate spatially and temporally to work towards restoring bone structural integrity without scar formation [4, 5].

During fracture healing, the local fracture hematoma at a fracture site is rich in multiple cytokines, inflammatory factors, and many kinds of cells, which are helpful for bone and soft tissue repair [6]. Horst et al. [7] pointed out that there was evidential upregulation of the concentrations of interleukin-6 (IL-6) and interleukin-8 (IL-8) in fracture hematomas when compared with the serum values in a combined trauma pig model. According to previous studies, IL-6 and IL-8 obviously affect the osteoblastogenesis and osteoclastogenesis [7–10]. Interleukin-34 (IL-34), a newly discovered cytokine, shows capable of inducing pro-inflammatory cytokines and chemokines such as IL-6 and IL-8 [11]. Thus, its osteogenic capacity should be explored further. Through comprehensive proteomic analyses, it was found to be the second ligand for colony-stimulating factor-1 receptor (CSF-1R). It binds to CSF-1R and possesses similar characteristics to CSF-1 in promoting monocyte viability and osteoclast generation [12, 13]. In the last few years, IL-34 has been gaining interest as a possible mediator for inflammatory arthritis (IA) [14–21]. It was reported that recombinant mouse IL-34 could induce the formation of osteoclasts in vitro and reduce trabecular bone mass in vivo [16]. In CSF-1^op/op^ mice, the presence of osteoclast precursors in the spleen seems to be supported by IL-34 expressed in vascular endothelial cells [22]. In addition, a study conducted by Cheng et al. [23] pointed out that IL-34 contributes to the survival of osteoclast progenitors, and in vitro, it further promoted receptor activator of NF-κB ligand (RANKL)-induced osteoclasts via the JAK2/STAT3 pathway. Therefore, IL-34 may play a key role on osteoclastogenesis. In accordance with previous studies, the dynamic balance of bone metabolism continuously depends on the regulation between osteoclastic bone resorption and osteoblastic bone formation [24–26]. Nevertheless, the effects and molecular mechanisms of IL-34 on osteoblastogenesis remain unknown. Here, we present a study to describe the mechanisms underlying the role of IL-34 in osteogenesis in human bone marrow-derived mesenchymal stem cells (hBMSCs).

Bone marrow-derived mesenchymal stem cells (BMSCs), with self-renewal capabilities, are progenitors of osteoblasts, chondrocytes, and adipocytes, playing a key role in bone formation and remodeling for the healing of bone defects [27]. BMSCs not only differentiate into bone but also exert modulatory effects via a variety of mechanisms to promote bone healing [28]. As such, they represent an important source for potential therapeutic use [29]. Meanwhile, many cytokines and growth factors can affect the differentiation of BMSCs and improve the migration and homing of BMSCs for bone regeneration [30–32]. Recently, several studies noted that an inflammatory microenvironment can potentially increase the immunogenicity of BMSCs and decrease BMSC viability and differentiation capacity [3, 8, 31].

The main goal of this research was to reveal a possible role of low-dose IL-34 in the molecular mechanisms on osteoblastogenesis and osteoclastogenesis. Our results founded that low-dose IL-34 regulates osteogenesis of hBMSCs partly via the PIK/AKT and ERK signaling pathway and enhances fracture healing, with neither promoting nor preventing osteoclastogenesis in vitro and osteoporosis in vivo.

## Materials and methods

### Cell isolation, culture conditions, reagents, and antibodies

hBMSCs were provided by Cyagen Biosciences (Guangzhou, China), and it was confirmed as having the potential to induce the differentiation of osteoblasts, chondrocytes, and adipocytes. The cells were incubated in hBMSC growth medium (Cyagen Biosciences, Guangzhou, China) at 37 °C in a cell incubator containing 5% CO_2_ with the medium being replaced every 3 days. Cells were trypsinized and passaged at nearly 80–90% confluence, and only passages three to seven were cultured in the follow-up experiments.

Six- to eight-week-old male C57BL/6 mice were used for the isolation of primary murine bone monocyte/macrophage precursors as described [33]. Cells were differentiated into bone marrow-derived macrophages (BMMs) in complete Minimum essential medium Eagle Alpha modification (α-MEM, Gibco) containing 30 ng/ml of macrophage colony-stimulating factor (M-CSF) for 3–4 days at 37 °C in a cell incubator with 5% CO_2_, and the medium was replaced every 2 days.

Recombinant human IL-34 (rhIL-34) was purchased from Novus Biologicals (CO, USA). Recombinant murine M-CSF and recombinant murine RANKL were purchased from Novoprotein Biotechnology (Shanghai, China). A phospho-p44/42 MAPK (ERK1/2) inhibitor (U0126; Selleck Chemicals) and a phospho-Akt inhibitor (MK-2206 2HCL; Selleck Chemicals) were used in this study. Specific antibodies against glyceraldehyde-3-phosphate dehydrogenase (GAPDH), runt-related transcription factor 2 (RUNX2), collagen type I a 1 chain (COL1A1), extracellular signal-regulated kinase 1/2 (ERK1/2), phospho-ERK1/2, Phospho-P38 MAPK, P38 MAPK, Phospho–NF-κB P65, NF-κB P65, Non-phospho (active) β-catenin, β-catenin, protein kinase B (AKT), Phospho-AKT, Phospho-IκBα, IκBα, Phospho-SAPK/JNK, SAPK/JNK, Nuclear factor of activated T cells cytoplasmic 1 (NFATc1/NFAT2), and C-Src were purchased from Cell Signaling Technology (Danvers, MA, USA). Specific antibodies against C-Fos and Cathepsin K were obtained from Abcam (Cambridge, UK). Specific antibodies against PGC1β and IL-34 were obtained from Santa Cruz Biotechnology (Dallas, TX, USA).

### Cytotoxicity assay

To evaluate the effects of IL-34 on the proliferation of hBMSCs and mice bone marrow-derived macrophages (mBMMs), a 96-well plate was applied to culture the cells with a density of 5 × 10^3^ cells/well and 8 × 10^3^ cells/well in triplicate, individually. After a 24-h period for adhesion, different concentrations of IL-34 (0, 0.0001, 0.001, 0.01 ng/ml) were performed for 1, 3, 5, or 7 days. Afterward, the medium was changed, and 10 μl Cell Counting Kit-8 (CCK-8, Dojindo, Kumamoto, Japan) buffer was added to each well, which was cultured for another 4 h at 37 °C. The optical density was measured on an ELX800 absorbance microplate reader (ELX808; BioTek, Winooski, VT, USA) at the wavelength of 450 nm (650 nm as reference).

### Osteoblastogenesis assay

For the determination of osteoblast differentiation in vitro, 12-well cell culture plates were applied to culture hBMSCs with a density of 3 × 10^4^/cm^2^ at 37 °C with 5% CO_2_. After 3 days, the cells were incubated in osteogenic differentiation medium (ODM; Dulbecco’s modified Eagle’s medium; 10% fetal bovine serum, 100 nM dexamethasone, 10 mM β-glycerophosphate, 1% penicillin-streptomycin and 0.05 mM l-ascorbic acid-2-phosphate) with different concentrations of IL-34 (0, 0.0001, 0.001, 0.01 ng/ml). Cells with ODM only were regarded as controls, and the medium of the treated cells was changed every 3 days.

Three days later, alkaline phosphatase (ALP) staining was performed. The cells were first fixed with 4% paraformaldehyde for 15 min, washed twice by phosphate buffer saline (PBS), and washed again but with double-distilled water (ddH_2_O) every 3 min for three times. Then, the cells were stained with the BCIP/NBT ALP color development kit (Beyotime, Shanghai, China). In accordance with the manufacturer’s instructions, ALP activity was calculated by the ALP Assay Kit (Beyotime, Shanghai, China) after the cells were lysed by lysis buffer including 1% Triton X-100, 20 mM Tris–HCl (pH 7.5), and 150 mM NaCl. Finally, the ALP activity was calculated at 405 nm by a microplate reader (ELX808; BioTek).

After inducting osteogenic differentiation for 12 days, Alizarin Red Staining (ARS) Kit (Cyagen, Guangzhou, China) was used for staining. The cells were fixed with 4% paraformaldehyde for 15 min after washed twice by PBS, and then washed with ddH_2_O every 3 min for three times before being stained with Alizarin Red S solution (Cyagen Biosciences, Guangzhou, China) for 30 min at room temperature. Afterward, the mineral deposition was observed and photographed using an inverted microscope with a digital camera. The stain was then absorbed by incubation with 10% cetylpyridinium chloride (MilliporeSigma, Billerica, MA, USA) for 1 h, and the solutions were plated on a 96-well plate with 200 μl/ well. The optical density values at 560 nm of the microplate reader (ELX808; BioTek) determined the total protein concentration.

### Osteoclastogenesis assay

To determine the osteoclast differentiation in vitro, mBMMs were seeded into 96-well plates (8 × 10^3^ cells/well) in triplicate with osteoclastogenic medium (complete α-MEM with 30 ng/ml M-CSF and 100 ng/ml RANKL) and various concentrations of IL-34 (0, 0.0001, 0.001, 0.01 ng/ml). Cells without treatment were regarded as controls. Cells with complete α-MEM (30 ng/ml of IL-34 and 100 ng/ml RANKL) were used to check our IL-34 worked. In the process of cultivation, the medium was changed every 2 days. Afterwards, cells were washed twice with PBS, fixed in 4% paraformaldehyde for 15 min, washed twice with PBS again, and stained for tartrate-resistant acid phosphatase (TRAP) staining (Sigma-Aldrich, Hannover, Germany), according to the manufacturer’s instructions. TRAP-positive cells with no less than two nuclei were considered as mature osteoclasts. The number and spread area of mature osteoclasts were measured by ImageJ software (National Institutes of Health, Bethesda, MD, USA).

### RNA extraction and quantitative RT-PCR

Gene expression levels of osteoclast and osteoblast formation were measured by quantitative RT-PCR (qRT-PCR). hBMSCs (3 × 10^4^ cells/cm^2^) and mBMMs (1 × 10^5^ cells/well) were seeded in six-well plates and cultured in the medium. The isolation and measurement of total cellular RNA was performed using the RNAiso reagent (Takara Bio, Kusatsu, Japan) and NanoDrop 2000. The absorbance of the samples at 260 nm was calculated in accordance with the manufacturer’s instructions (Thermo Fisher Scientific, MA, USA). Total RNA (#1 μg) was reverse transcribed into complementary DNA (cDNA) in a 20 μl reaction volume (Takara). Two microliters cDNA was used as the template with Power SYBR® Green PCR Master Mix (Takara), and qRT-PCR was performed in triplicate by the ABI StepOnePlus System (Thermo Fisher Scientific). As housekeeping genes, 18S or β-actin was used, and all of the reactions were repeated three times independently. Sangon Biotech (Shanghai, China) synthesized all of the primers used in this work. Primer sequences are listed in Table 1. The qRT-PCR reaction was as follows: 95 °C for 30 s, followed by 40 cycles of 95 °C for 5 s, and 60 °C for 30 s. The expression levels of all of the genes were evaluated by 2^−△△Ct^ method.

**Table 1:** Sequences of primers for qRT-PCR

Primers (5′–3′)		
Gene	Forward	Reverse
h-COL1A1	CAGATCACGTCATCGCACAAC	GAGGGCCAAGACGAAGACATC
h-RUNX2	TGGTTACTGTCATGGCGGGTA	TCTCAGATCGTTGAACCTTGCTA
h-OCN	CACTCCTCGCCCTATTGGC	CCCTCCTGCTTGGACACAAAG
h-SP7	AGCCCATTAGTGCTTGTAAAGG	CCTCTGCGGGACTCAACAAC
h-ALP	ACCACCACGAGAGTGAACCA	CGTTGTCTGAGTACCAGTCCC
h-OPN	CTCCATTGACTCGAACGACTC	CAGGTCTGCGAAACTTCTTAGAT
h-18S	CCAGACAAATCGCTCCACCAAC	GACTCAACACGGGAAACCTCAC
m-β-ACTIN	TCTGCTGGAAGGTGGACAGT	CCTCTATGCCAACACAGTGC
m-NFATc1	CGTTGCTTCCAGAAAATAACA	TGTGGGATGTGAACTCGGAA
m-CATHEPSIN K	CTTCCAATACGTGCAGCAGA	TCTTCAGGGCTTTCTCGTTC
m-C-FOS	CCAGTCAAGAGCATCAGCAA	AAGTAGTGCAGCCCGGAGTA
m-CTR	TGCTGGCTGAGTGCAGAAACC	GGCCTTCACAGCCTTCAGGTAC
m-MMP-9	CAAAGACCTGAAAACCTCCAA	GGTACAAGTATGCCTCTGCCA

*h* human, *m* mice

### Western blotting analyses

To investigate the effects of IL-34 on multiple signaling pathways, hBMSCs (3 × 10^4^ cells /cm^2^) and mBMMs (5 × 10^5^ cells/well) were seeded in six-well plates and cultured in the medium. Total lysates of cells were extracted by lysis for 30 min on ice in a ripa buffer containing with phosphatase and protease inhibitor cocktails (Beyotime). The centrifugation to clear the lysates and collect the supernatants was set at 14,000 rpm for 10 min at 4 °C. After electrophoresis, the SDS polyacrylamide gel was transferred to a polyvinylidene fluoride (PVDF) membrane (MilliporeSigma), which was then probed with the primary antibodies. Then, membranes were blocked with 10% non-fat milk and 0.1% Tween in tris-buffered saline for 1 h at room temperature. Afterward, the membranes were incubated at 4 °C overnight with primary antibodies. After washing with 0.1% Tween in tris-buffered saline for three times and incubation with horseradish peroxidase-conjugated secondary antibodies (anti-mouse or anti-rabbit; Beyotime) for 1 h at room temperature, proteins were visualized using an enhanced chemiluminescent detection reagent (MilliporeSigma) and an XRS chemiluminescence detection system (Bio-Rad Laboratories, Hercules, CA, USA).

### Immunofluorescence assay

A 12-well plate was used to place hBMSCs in induction medium, and a fluorescence microscope (EU5888; Leica Camera, Wetzlar, Germany) was used for the evaluation of RUNX2 and COL1A1. At room temperature, the cells were treated with 4% paraformaldehyde. After 15 min, hBMSCs were permeabilized for 5 min with 0.1% Triton X-100 in PBS and blocked in 2% bovine serum albumin for 30 min. Fixed cells were washed and incubated overnight with anti-RUNX2 (1:500; CST) and anti-COL1A1 (1:500; CST). Then the fluorescence-conjugated secondary antibody (Beyotime) was added to the cells for 60 min, and DAPI (Nanjing KeyGen Biotech, Nanjing, China) was used to stain the nuclei for 5 min. The images were captured by a fluorescence microscope (Leica Camera) and the fields were selected randomly.

### Lentiviral packaging and cell infection

A lentiviral package was applied by Cyagen Biosciences (Guangzhou, China), including lentiviral particles to overexpress IL-34 (IL-34 overexpressed (OE) group) and lentiviral GFP particles (the negative control (NC) group). hBMSCs (3 × 10^4^ cells/cm^2^) were seeded in six-well plates and cultured in the medium. When hBMSCs reached at 30–50% confluence, cells were cultured in lentiviral particles together with 5 μg/ml polybrene in the growth medium, in accordance with the manufacturer’s instructions. The GFP fluorescence was regarded as the efficiency of transduction and the culture medium was replaced 12 h later. When at a confluence of 80–90%, the cells were passaged and used the experiments here described. The qRT-PCR and Western blotting analyses were performed to determine the difference in osteo-specific genes and proteins.

### ELISA

An ELISA (Mskbio, Wuhan, China) was used to evaluate the concentrations of IL-34 in the culture medium of both OE and NC. After the cells were infected with the lentiviral package and then cultured for 24 h, the medium was collected to measure the concentrations of IL-34, according to the manufacturer’s instructions.

### In vivo evaluation in animals

This study was approved by the Institutional Animal Care and Use Committee of the Second Affiliated Hospital, School of Medicine, Zhejiang University (approval number: 2018-078). In accordance with the Animal Care and Use Committee guidelines of Zhejiang province together with the laboratory animals’ care and use guidelines, we performed the animal experiments. Thirty-six male Sprague Dawley rats (eight-week-old, 200 g) from the Academy of Medical Sciences of Zhejiang Province were used as tibial bone defect or ovariectomized (OVX) models [33]. Eighteen rats were separated equally and haphazardly into three groups (*n* = 6 per group): (1) Blank group: defects without treatment; (2) PBS group: defects treated with PBS (negative control group treated with PBS); and (3) IL-34 group: defects treated with local injection of IL-34 (20 μl IL-34 at 0.01 ng/ml). Briefly, rats were anesthetized by intraperitoneal injection with 0.3% pentobarbital sodium (Sigma) at 30 mg/kg body weight. After wiping the knee joint with alcohol, the closed reduction and internal fixation were performed by a 1.3-mm intramedullary fixation pin set into the tibial cavity. A 5 × 2 mm^2^ tibial defect was formed in all the rats nearly 7 mm from the proximal tibial growth plate by a grinding drill and penetrated through the cortex of the bone. The operation was performed on the same leg for each group. The incision was then sewed up with 4–0 absorbable sutures. Local injection with 20 μl IL-34 (0.01 ng/ml) was done in the tibial defect sites of the rats from IL-34 group every 2 days after operation, and the rats from rest groups were treated with (PBS group) or without (BLANK group) 20 μl PBS. The remaining 18 rats were randomized into three groups of six rats each: sham-surgery rats treated with PBS (BLANK group), OVX rats treated with PBS (OVX group), and OVX rats treated with IL-34 (OVX + IL-34 group). One week after ovariectomy, IL-34 (0.1 μg/kg) or PBS was injected intraperitoneally into each OVX rat every 2 days. After 2 weeks and 8 weeks, the rat tibial bone defects model and the OVX rat model were euthanized using excess anesthesia, respectively. No deaths or side effects occurred during the intervention. The tibia from each rat was collected and scanned by microcomputed tomography (Micro-CT). Specimens from the rat tibial bone defects model were fixed in 4% paraformaldehyde. One day later, they were decalcified by 10% ethylene diaminetetra acetic acid (EDTA, Sigma) with 0.1 M PBS (pH 7.4) for 8 weeks, with a mixture change per week. After decalcification, specimens were embedded in paraffin, and sections of the proximal tibias were obtained for H&E, SO/FG, and Masson’s trichrome analysis.

### Micro-CT and bone histomorphometric analyses

The tibias were analyzed by a Micro-CT (Scanco Medical, Brüttisellen, Switzerland) instrument with scanning method set at an isometric resolution of 14.8 μm with an exposure time of 300 ms. The X-ray energy settings were 70 kV and 80 μA. Trabecular bone volume per total volume (BV/TV), trabecular bone surface per bone volume (BS/BV), mean trabecular thickness (Tb.Th), mean trabecular number (Tb.N), mean trabecular separation (Tb.Sp), and mean connectivity density (Conn-Dens) were quantified to evaluate the microstructure of the tibias.

### Statistical analysis

Results are expressed as means ± SD. SPSS software (v.22.0; IBM, Armonk, NY, USA) was used to perform the statistical analyses. All of the experiments were independently accomplished no less than three times. Statistical differences were evaluated by two-tailed Student’s *t* test or one-way ANOVA with Bonferroni’s post hoc test. A two-way ANOVA with Bonferroni multiple comparisons post hoc test was used in the comparison of the treated groups at different time points. A *P* ≤ 0.05 was regarded as being significantly different.

## Results

### The effects of low-dose IL-34 on hBMSC and mBMM viability

**Fig. 1:**
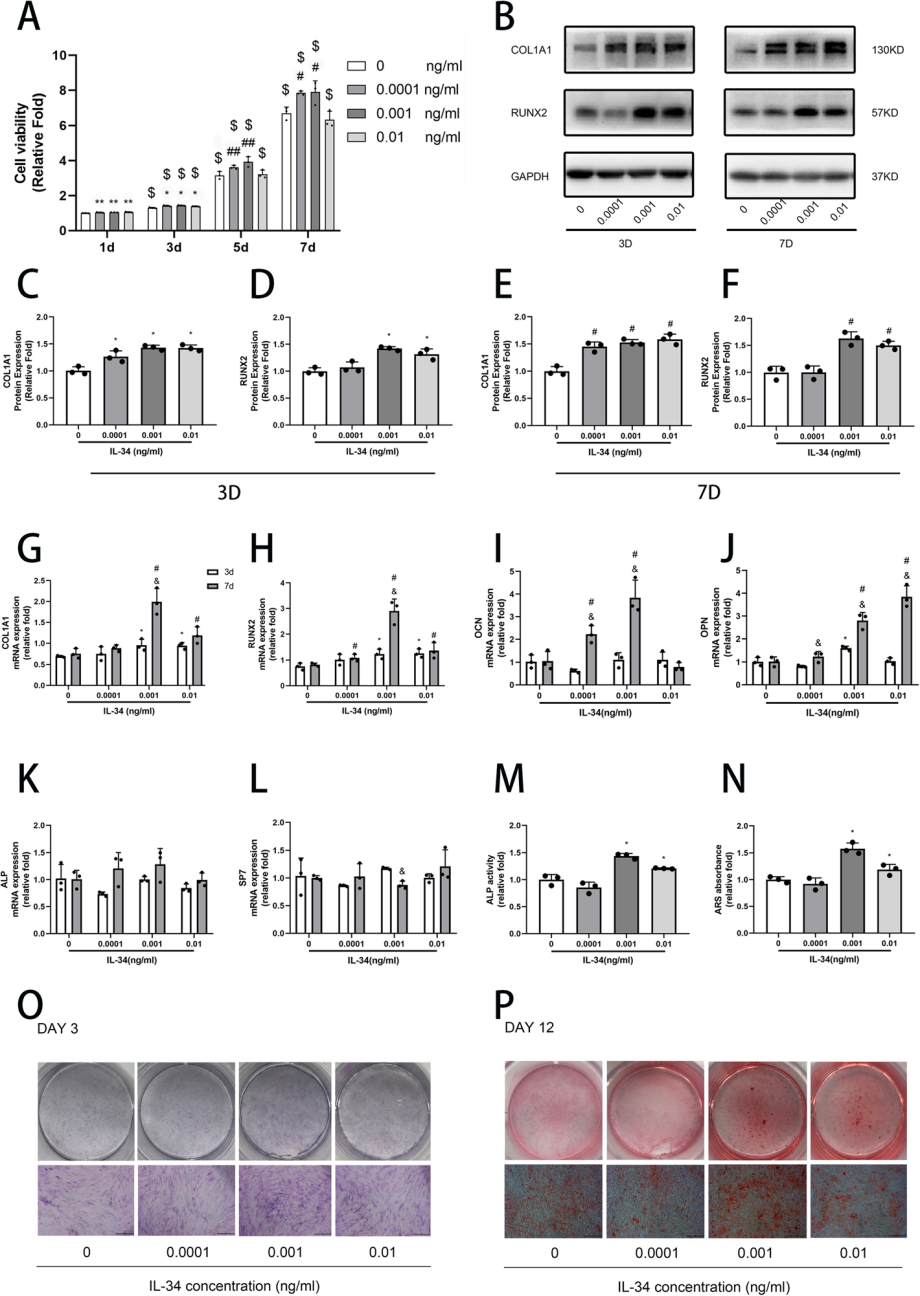
The effect of low-dose IL-34 on viability and osteoblastogenesis in hBMSCs. **a** A CCK-8 was used to examine the viability after cells cultured with low-dose IL-34 for 1, 3, 5, and 7 days. **b–f** The expression of RUNX2 and COL1A1 proteins were evaluated by Western blotting analysis after osteogenic differentiation for 3 and 7 days. **g–l** The expression of RUNX2, COL1A1, OCN, ALP, SP7, and OPN mRNA was determined by qRT-PCR on days 3 and 7 after osteogenic differentiation. **m, o** ALP staining and ALP activity were performed after osteogenic differentiation for 3 days. **n, p** ARS and ARS absorbance were performed after osteogenic differentiation for 12 days. All of the experiments were independently accomplished no less than three times. Data are means ± SD. ^**^P < 0.05 vs. the control group on day 1, ^*^P < 0.05 vs. the control group on day 3, ^##^*P* < 0.05 vs. the control group on day 5, ^#^*P* < 0.05 vs. the control group on day 7, ^@^*P* < 0.05 vs. the control group on day 12, ^$^*P* < 0.05 vs. the 1 day group at the same concentration, ^&^*P* < 0.05 vs. the 3 days group at the same concentration. Scale bars, 500 μm

The effects of low-dose IL-34 on hBMSC viability at days 1, 3, 5, and 7 are shown in Fig. 1a. Cells treated with IL-34 from 0.0001 to 0.01 ng/ml, the viability rate of hBMSCs increased, except for the 0.01 ng/ml at day 7 condition. The effects of IL-34 on mBMM viability are depicted in Figure S1.

### Low-dose IL-34 promoted osteoblastogenesis in hBMSCs

To evaluate the role of low-dose IL-34 on bone formation, the expression of RUNX2 and COL1A1 were measured by qRT-PCR and Western blotting analysis. Meanwhile, the expression of osteocalcin (OCN), ALP, osteopontin (OPN), and zinc finger transcription factor (SP7/Osterix) were determined by qRT-PCR. IL-34 increased the expression of RUNX2 (days 3 and 7) and COL1A1 (days 3 and 7) between 0.0001 and 0.01 ng/ml except the expression of RUNX2 (days 3 and 7) at 0.0001 ng/ml (Fig. 1b–f). The qRT-PCR analysis revealed that RUNX2 and COL1A1 mRNA expression was increased by 0.001 and 0.01 ng/ml IL-34 on day 3 and 7 (Fig. 1g–h). OCN expression was increased by IL-34 at 0.0001 to 0.001 ng/ml on day 7 (Fig. 1i). OPN mRNA expression was increased by IL-34 at 0.0001 to 0.01 ng/ml on day 7 and 0.001 ng/ml on day 3 (Fig. 1j). However, there were no significant differences between ALP and SP7 expression levels under 0.0001 to 0.01 ng/ml doses on days 3 and 7 when compared with the control group (Fig. 1k–l). IL-34 positively regulated ALP activity and calcium deposit formation at 0.001 and 0.01 ng/ml (Fig. 1m–p).

### Low-dose IL-34 enhanced osteogenic differentiation of hBMSCs partly via the PI3K/AKT and ERK signaling pathways

**Fig. 2:**
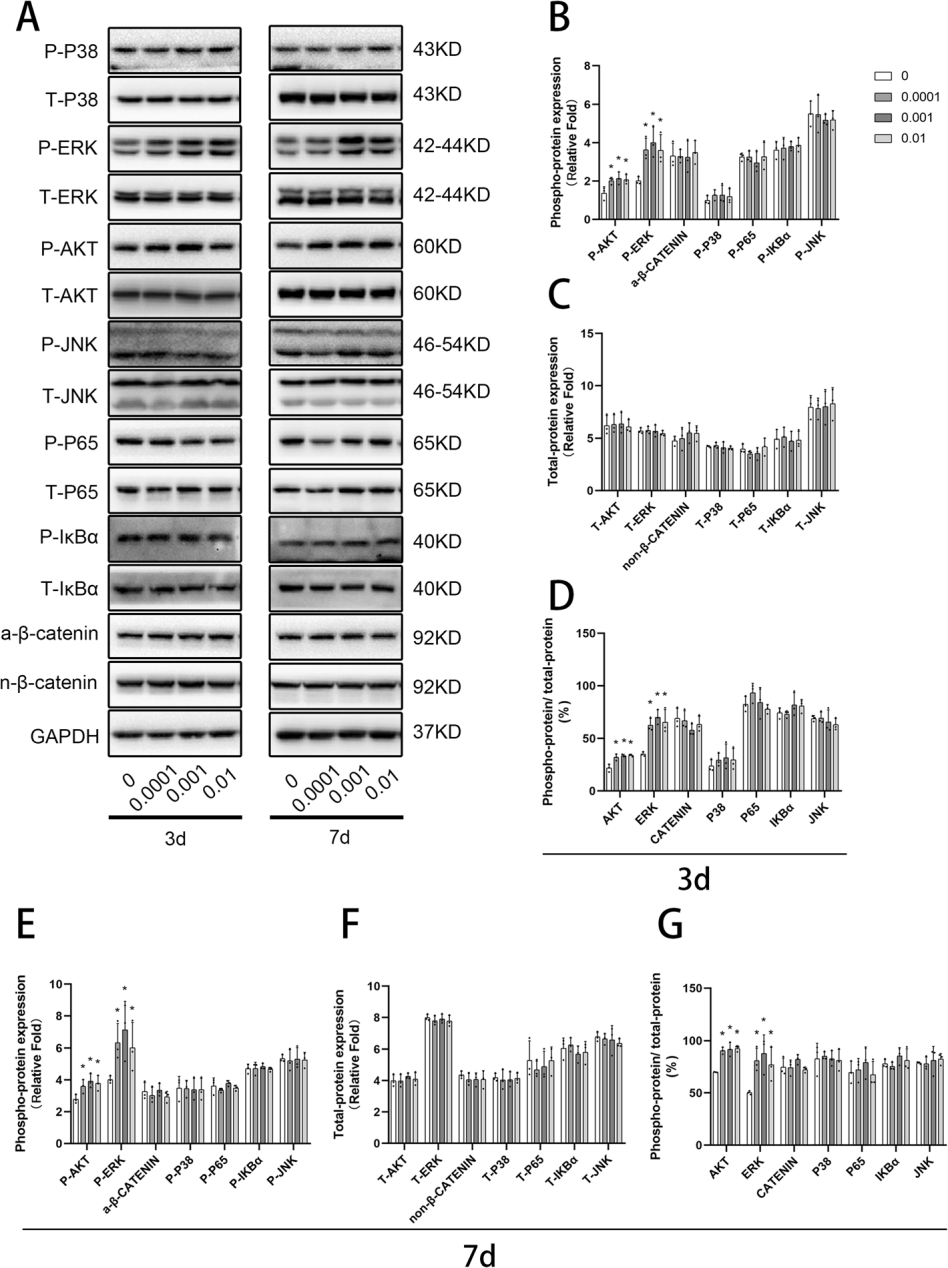
Low-dose IL-34 prompts activation of PI3K/AKT and ERK signaling during osteoblastogenesis in hBMSCs. **a–g** Western blotting analysis on days 3 and 7 was performed to determine the expression of NF-κB, MAPK, PI3K/AKT, and Wnt/β-catenin signaling pathways proteins on osteoblastogenesis. All of the experiments were independently accomplished no less than three times. Data are means ± SD. ^*^P < 0.05 vs. the control group on day 3, ^#^*P* < 0.05 vs. the control group on day 7, ^&^*P* < 0.05 vs. the 3 days group at the same concentration

To investigate the exact mechanism by which IL-34 positively regulated osteoblastogenesis, Western blotting was applied to measure whether IL-34 modulated the activation of the NF-κB, MAPK, PI3K/AKT, and Wnt/β-catenin signaling pathways, and the results were quantified (Fig. 2a–g). The phosphorylation level of AKT and ERK increased significantly after IL-34 treatment at all of the concentrations on days 3 and 7 when compared with the control group (Fig. 2b, e). However, the phosphorylation levels of the other proteins were not affected by IL-34 (Fig. 2b, e). The total levels of all proteins showed no significant difference among different groups (Fig. 2c, f). The phospho-protein/total protein of AKT and ERK were significantly different between the treated groups and control group (Fig. 2d, g). To further elucidate the effects of the PI3K/AKT and ERK signaling pathways on the regulation of hBMSC osteoblastogenesis by IL-34, the IL-34-induced inhibitory effects of these two pathways on bone formation were analyzed.

**Fig. 3:**
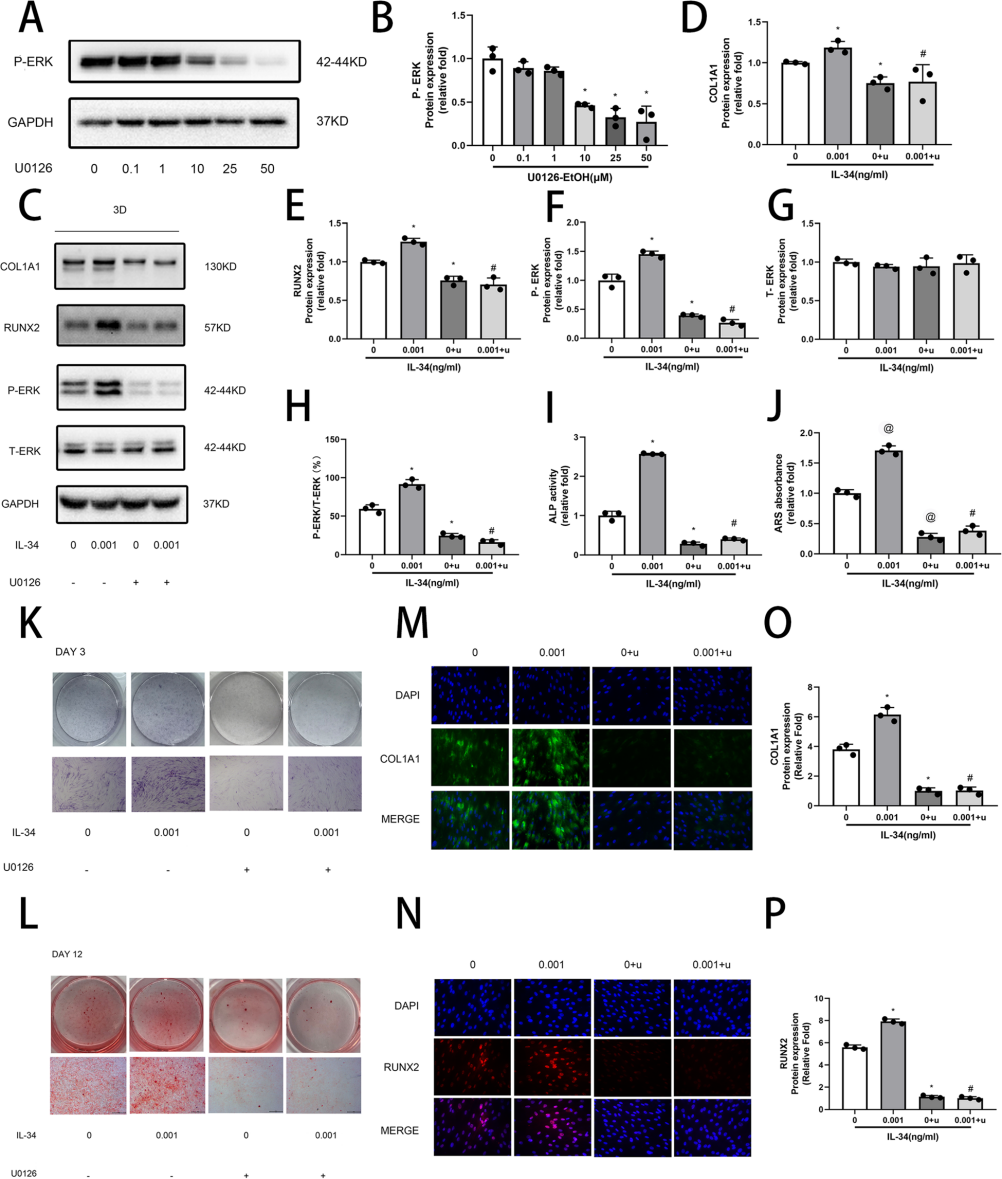
Inhibitor specific for ERK suppresses the osteogenic differentiation caused by low-dose IL-34. **a, b** Determination of suitable concentration of inhibitor. **c–h** Expression of COL1A1, RUNX2, and ERK signaling pathway proteins was determined by Western blotting analysis on day 3 of osteogenesis. **i, k** ALP staining and activity on day 3 of osteogenesis. **j, l** ARS and quantitation on day 12 of osteogenesis. **m, o** Expression of COL1A1 proteins was determined by immunofluorescence on day 3 of osteogenesis. **n, p** Expression of RUNX2 proteins was determined by immunofluorescence on day 3 of osteogenesis. All of the experiments were independently accomplished no less than three times. Data are means ± SD. ^*^P < 0.05 vs. the control group on day 3, ^@^*P* < 0.05 vs. the control group on day 12, ^#^*P* < 0.05 vs. the IL-34 (0.001 ng/ml) group. Scale bars, ALP staining and ARS, 500 μm. Immunofluorescence, 25 μm

As Fig. 3a, b shows, there was a significant difference between the control group without U0126 and treated groups with U0126 at 10, 25, and 50 μM respectively when hBMSCs cultured in the ODM during osteogenic differentiation. We chose 25 μM as the U0126 concentration in the following experiment. The increased expression of COL1A1, RUNX2, and P-ERK caused by the IL-34 (0.001 ng/ml) treatment was significantly inhibited when the cells were cultured with U0126 for 3 days (Fig. 3c–f), whereas there was no difference among these groups in the expression of T-ERK (Fig. 3g). The P-ERK/T-ERK was significantly decreased when cells were cultured with U0126 (Fig. 3h). A similar decrease was observed in the ALP activity and ARS following the addition of U0126 for 3 days (Fig. 3i-l).

**Fig. 4:**
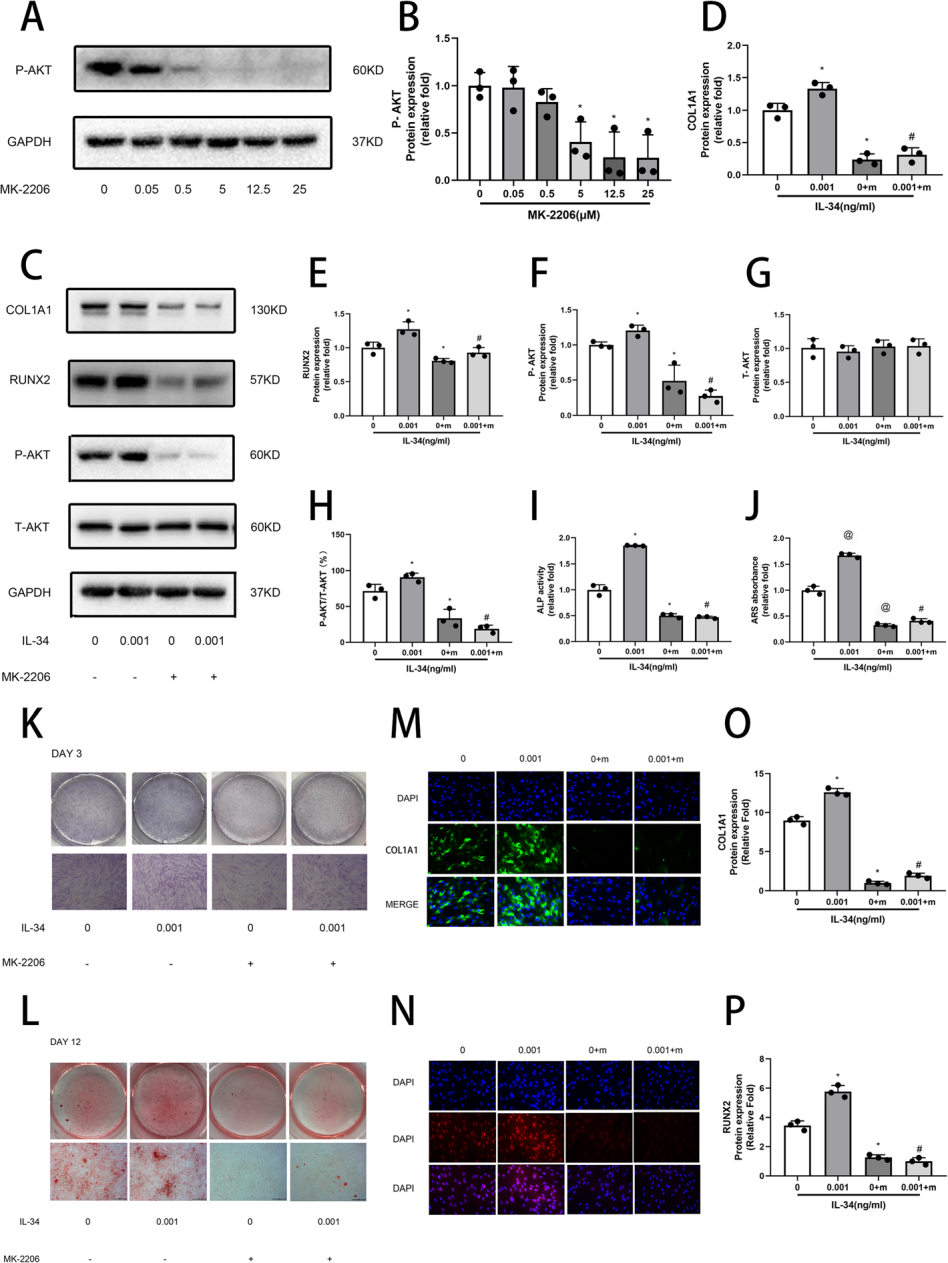
Inhibitor specific for PI3K/AKT suppresses the osteogenic differentiation caused by low-dose IL-34. **a, b** Determination of suitable concentration of inhibitor. **c–h** Expression of COL1A1, RUNX2, and PI3K/AKT signaling pathway proteins was determined by Western blotting analysis on 3 d of osteogenesis. **i, k** ALP staining and activity on day 3 of osteogenesis. **j, l** ARS and quantitation on day 12 of osteogenesis. **m, o** Expression of COL1A1 proteins was determined by immunofluorescence on day 3 of osteogenesis. **n, p** Expression of RUNX2 proteins was determined by immunofluorescence on day 3 of osteogenesis. All of the experiments were independently accomplished no less than three times. Data are means ± SD. ^*^P < 0.05 vs. the control group on day 3, ^@^*P* < 0.05 vs. the control group on day 12, ^#^*P* < 0.05 vs. the IL-34 (0.001 ng/ml) group. Scale bars, ALP staining and ARS, 500 μm. Immunofluorescence, 25 μm

Cells treated with MK-2206 (5, 12.5, and 25 μM) were significantly different from the control group (Fig. 4a, b). Five micromolars of MK-2206 was the concentration used in the follow-up work. There was a great decrease among the COL1A1, RUNX2, and P-AKT expression levels after the cells were incubated with MK-2206 for 3 days (Fig. 4c–f). However, the expression of T-AKT did not change (Fig. 4g). The P-AKT/T-AKT was significantly decreased when cells were cultured with MK-2206 (Fig. 3h). ALP activity and ARS performed a similitude consequence after treatment with MK-2206 (Fig. 4i–l).

An IF assay was performed to further assess the effects of the PI3K/AKT and ERK signaling pathways. Higher COL1A1 and RUNX2 expression levels were observed in the IL-34 treatment group relative to the control group, and the levels of both were higher than in the groups treated with inhibitors (Fig. 3m–p and Fig. 4m–p).

### The effects of endogenously overexpressed IL-34 on osteogenesis and the expression of osteoblast-related genes and proteins in vitro

**Fig. 5:**
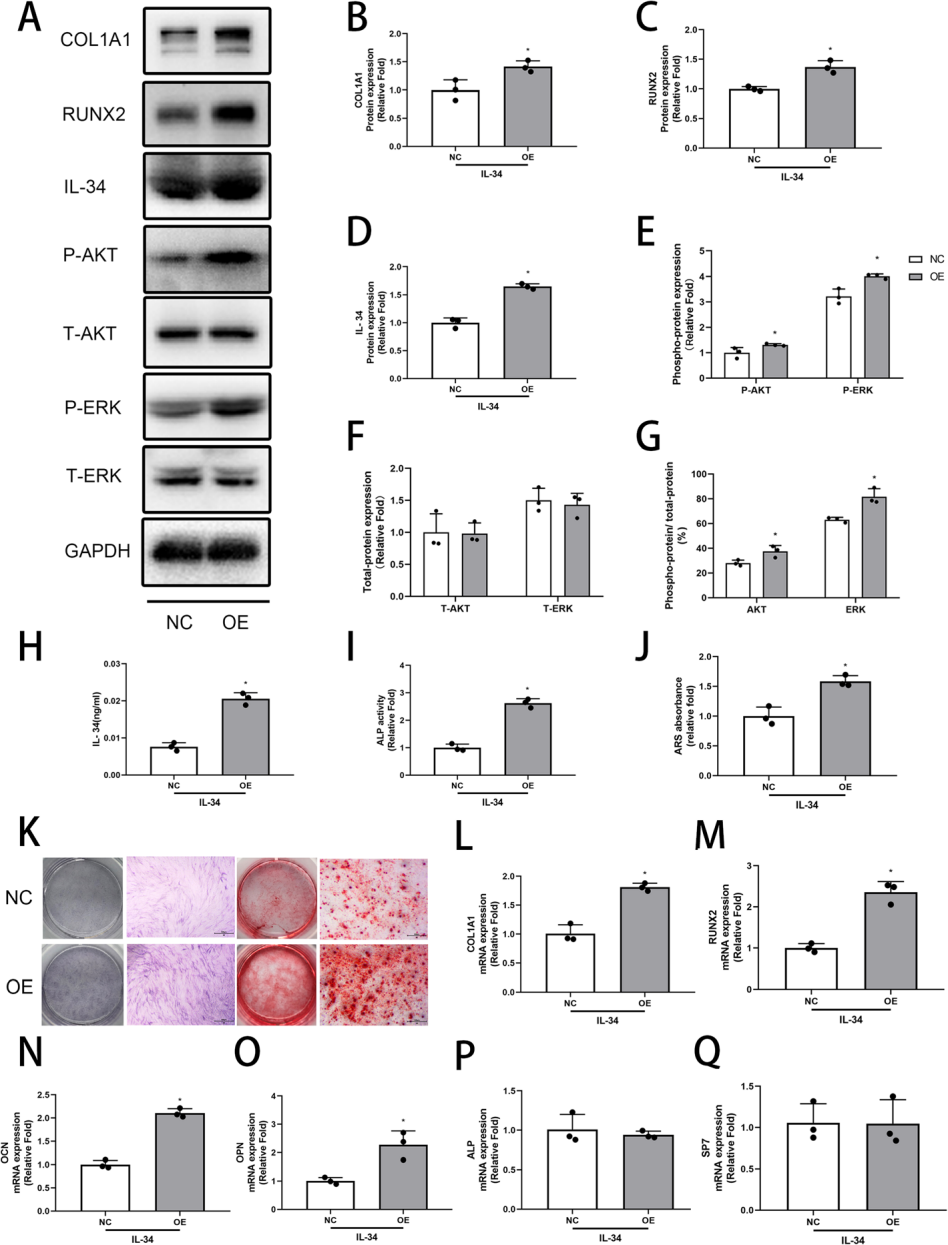
The effects of IL-34 overexpression hBMSC cell line on osteogenic differentiation. **a–g** Expression of RUNX2, COL1A1, IL-34, P-AKT, T-AKT, P-ERK, and T-ERK was determined by Western blotting on day 3 of osteogenesis. **h** The results of ELISA. **i-k** ALP staining and activity on day 3 and ARS and quantitation on day 12 of osteogenesis, respectively. **l–q** The mRNA expression levels of COL1A1, RUNX2, OCN, OPN, ALP, and SP7 were evaluated by qRT-PCR. All of the experiments were independently accomplished no less than three times. Data are means ± SD. ^*^P < 0.05 vs. the control group on day 3, ^@^*P* < 0.05 vs. the control group on day 12. Scale bars, 500 μm

A IL-34 overexpression hBMSC cell line was constructed using a lentiviral vector to demonstrate the effects of endogenous IL-34 further. After infection, the cells were screened for GFP fluorescence. Three days later, the expression levels of COL1A1, RUNX2, P-ERK, T-ERK, P-AKT, T-AKT, and IL-34 were determined by Western blotting analyses. Measured up against the control group, all of the protein levels were significantly increased, except T-ERK and T-AKT (Fig. 5a–f). The P-ERK/T-ERK and P-AKT/T-AKT were significantly increased (Fig. 5g). The expression levels of osteo-specific genes were assessed by qRT-PCR. COL1A1, RUNX2, OCN, and OPN were significantly increased in the OE group (Fig. 5l–o). However, there was no difference in ALP and SP7 (Fig. 5p–q). The results of ELISA, ALP activity, and ARS further confirm these above conclusions (Fig. 5h–k).

### Addition of exogenous low-dose IL-34 accelerated bone healing in a rat tibial osteotomy model

**Fig. 6:**
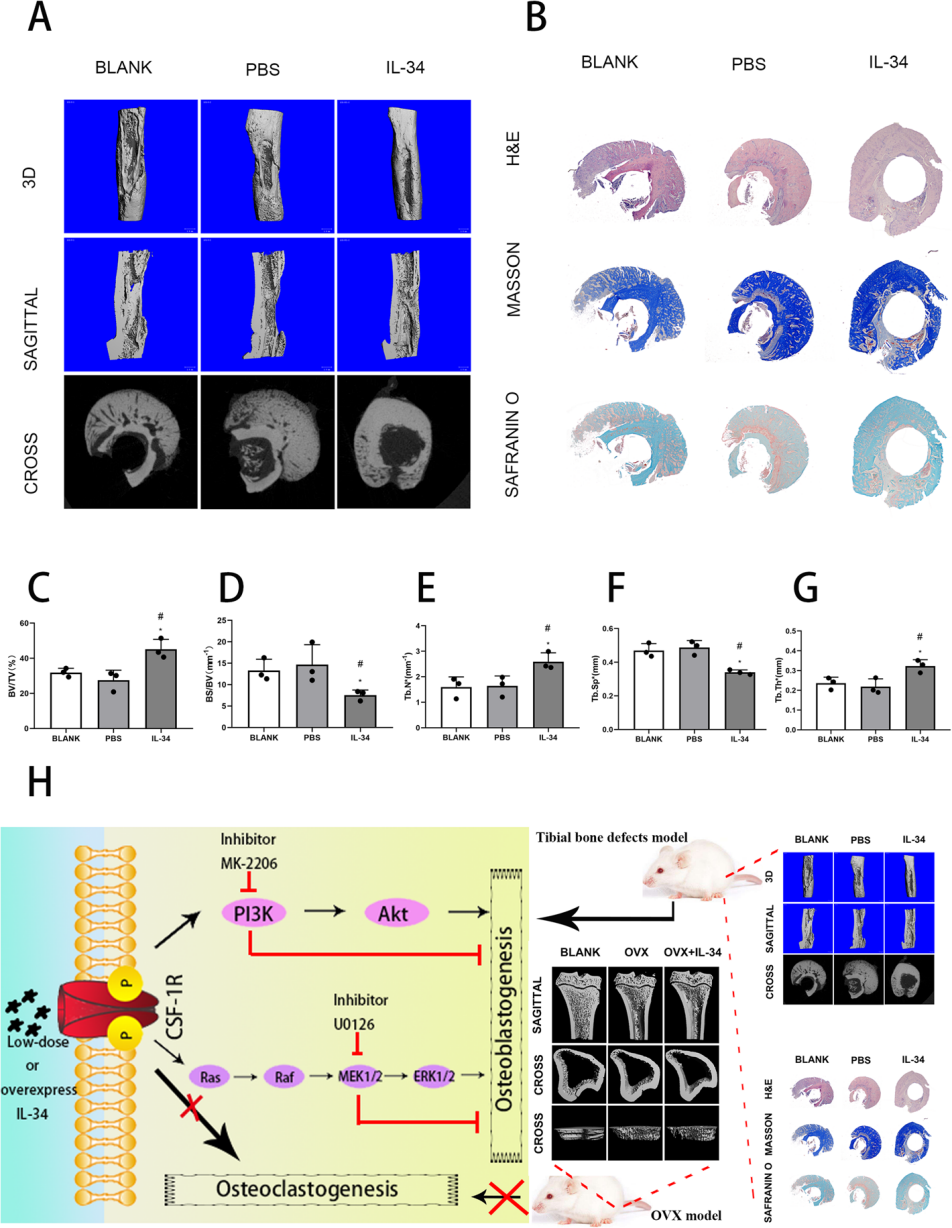
Exogenous IL-34 enhanced bone recovering in a rat tibial osteotomy model. **a** Micro-CT analysis for bone healing. **b** Histological analysis for bone healing. H&E, hematoxylin and eosin staining. Masson, Masson’s trichrome staining. Safranin O, Safranin O and fast green. **c–g** BV/TV, BS/BV, Tb.Th, Tb.N, and Tb.Sp values are presented. **h** Schematic representation of the experiments presented in this figure. All of the experiments were independently accomplished no less than three times. Data are means ± SD. ^*^P < 0.05 vs. the BLANK group, ^#^*P* < 0.05 vs. the PBS group. Scale bars, 1 mm

To complement the previous experiment, the effect of an exogenous low-dose of IL-34 on bone healing was performed. After 2 weeks, micro-CT analysis revealed that the bone defect was present legibly in the blank and PBS groups (Fig. 6a). In the IL-34 group, this gap was obscured, and more bridging callus formation was significantly presented in the operation area compared to the blank and PBS groups. Quantitatively, by contrasted with the blank and PBS group, there was an evident increase of fractures in the IL-34 group in the BV/TV, BS/BV, Tb.Th, Tb.N, and Tb.Sp (Fig. 6c–g). Histological analysis revealed that there was less bridging bone formed in the operated area in the blank and PBS groups, and a better cortex callus formation was observed in the IL-34 group in contrast with the rest groups (Fig. 6b).

### Low-dose IL-34 has no effect on osteoclastogenesis

**Fig. 7:**
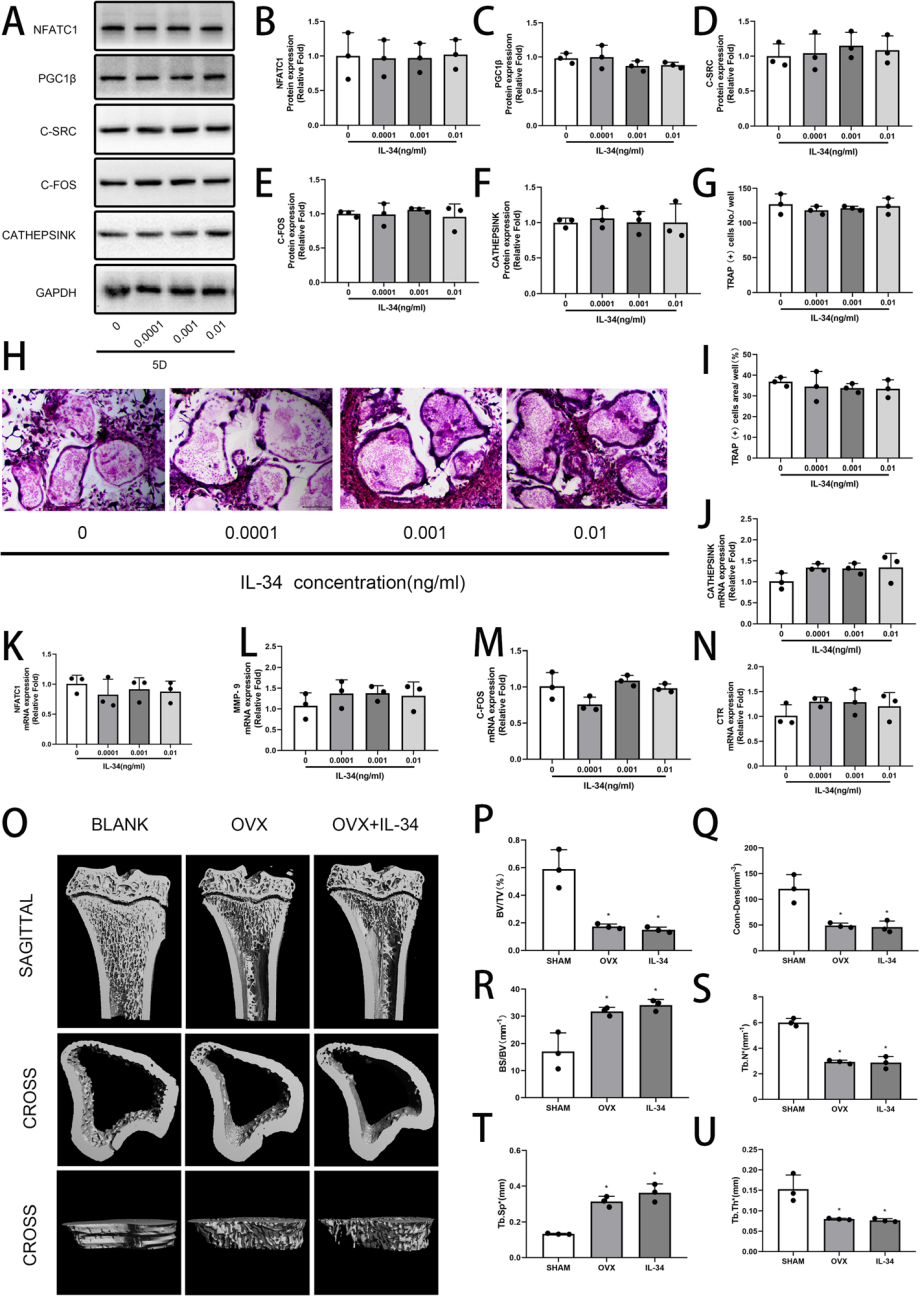
Low-dose IL-34 has no effect on osteoclastogenesis. **a–f** The expression of NFATc1, PGC1β, C-SRC, C-FOS, and Cathepsin K was determined by Western blotting on day 5 of osteoclastogenesis has no difference among various concentrations of low-dose IL-34. **g–i** mBMMs were cultured with various concentrations of low-dose IL-34 in the presence of M-CSF (30 ng/ml) and RANKL (100 ng/ml). **j–n** The mRNA expression levels of Cathepsin K, NFATc1, MMP-9, CTR, and C-FOS evaluated by qRT-PCR have no difference among various concentrations of low-dose IL-34. **o** Micro-CT reconstruction images from the 3 groups at 8 weeks after surgery. **p–u** BV/TV, BS/BV, Tb.Th, Tb.N, Tb.Sp, and Conn-Dens values are presented. All of the experiments were independently accomplished no less than three times. Data are means ± SD. ^*^P < 0.05 vs. the control group. Scale bars, 1 mm

The expression of osteoclast-related genes and proteins was not affected by the exogenous low-dose IL-34 in the experimental groups in contrast with the control group (Fig. 7a–f, j–n). In addition, the results of TRAP staining and micro-CT analysis of OVX rat models were in line with the conclusion (Fig. 7g–i, o–u). Thus, the low-dose IL-34 had no effect on osteoclast formation. Our IL-34 was confirmed worked by TRAP staining with a concentration of 30 ng/ml (Figure S2A-C). A simple diagram for this experiment is shown in Fig. 6h.

## Discussion

IL-34, the second ligand for CSF-1R, was identified on a functional screening of a library of proteins secreted by the embryonic kidney cell line transfected with recombinant cDNAs [12]. Although, structurally, it is markedly different from any other proteins, IL-34 binds to the CSF-1 receptor strongly and is similar to CSF-1 in the ability to enhance monocyte viability and osteoclast generation [12, 13, 32, 34]. In addition, IL-34 has been regarded as a promising clinical biomarker and therapeutic target for IA [14, 20, 21]. Studies using concentrations of IL-34 above 2.5 ng/ml have reported effects on the differentiation, proliferation, and survival of osteoclasts [13, 22, 23, 35]. However, the effect of IL-34 on the bone metabolism, particularly when low doses are used, has rarely been described. Similarly, IL-34 has not been linked to the effects of osteogenic differentiation in hBMSCs. Finally, there is no description to date of a role for IL-34 in the molecular mechanisms of the osteogenesis. Here, to our knowledge, we first revealed the underlying mechanisms of low-dose IL-34 on the regulation of bone homeostasis. IL-34 promoted the bone formation of hBMSCs partly via the PI3K/AKT and ERK signaling pathways in vitro. Meanwhile, a rat tibial bone defect model with a local injection of IL-34 produced a better recovery in vivo. However, low-dose IL-34 did not contribute to the differentiation of mBMMs in vitro, and a rat OVX model gave results consistent with this conclusion. These observations demonstrate that low-dose IL-34 enhances the osteogenic differentiation of hBMSCs, at least by partial activation of the PI3K/AKT and ERK signaling pathways, but it has no effect on osteoclastogenesis.

Serum IL-34 levels in healthy people were found to be 152.0 (92.0–234.0) pg/ml, 56.74 ± 2.30 pg/ml or 49.1 ± 78.5 pg/ml [13, 15–17, 22, 23]. Thus, in the present study, we regarded the concentration of IL-34 in a range from 0.0001 to 0.01 ng/ml as a low dose. Chen et al. revealed that IL-34 together with RANKL can induce the formation of murine osteoclasts from not only splenocytes but also bone marrow cells in a dose-dependent manner (2.5 ng/ml, 25 ng/ml, and 100 ng/ml), and these cells have bone resorption activity [13]. According to Nakamichi et al. IL-34 appears to play a pivotal role in the generation and storage of osteoclast precursors in the spleen and osteoclastogenesis in *CSF-1*^op/op^ mice [22]. A study conducted by Cheng et al. first demonstrated that IL-34 was conducive to the survival of osteoclast progenitors and further promoted RANKL-induced osteoclast formation by the JAK2/STAT3 pathway in vitro [23]. Furthermore, it has been reported that TNF-α upregulates osteoclastogenic cytokine IL-34 production through the activation of NF-κB and JNK signaling in the synovial cells of rheumatoid arthritis (RA) patients [35]. In the present study, we revealed that a low-dose IL-34 obviously heightened the expression of osteogenic-specific genes and proteins in hBMSCs, which filled a blank by showing that IL-34 plays an important role in osteogenesis. However, no significant differences were observed during osteoclastogenesis with the low-dose IL-34. This may be associated with the concentration of IL-34, which was too low to work for osteoclast formation both in vivo and in vitro. These results demonstrated that in low dose, IL-34 contribute to osteoblastogenesis rather than osteoclastogenesis.

Given that IL-34 has been demonstrated to play dominant roles in synovial inflammation and bone erosion, it possibly led to RA and osteoarthritis (OA) pathology [12]. Plenty of studies have concentrated on the underlying correlation between the concentrations of IL-34 in the circulation or joint fluid and clinical parameters of RA patients. A case-control study containing with 100 RA patients and 59 healthy controls not only measured serum IL-34 levels in RA patients and healthy controls but also observed that serum IL-34 levels were significantly greater in RA patients than in healthy controls (603.5 [123.3–1673.0] vs. 152.0 [92.0–234.0] pg/ml) [15]. These conclusions agreed with the results conducted by Wang et al., who pointed out that serum IL-34 levels in RA patients were markedly higher than in healthy controls (269.72 ± 14.71 pg/ml vs. 56.74 ± 2.30 pg/ml) [16]. The concentrations of IL-34 levels in serum were found to be correlated with several clinical variables [15, 16]. Moon et al. indicated that the serum IL-34 levels in RA patients were much higher than in OA patients and healthy controls. The mean serum IL-34 levels were 49.1 ± 78.5 pg/ml, 36.6 ± 38.0 pg/ml, and 188.0 ± 550.3 pg/ml in healthy controls, OA, and RA patients, respectively [17]. All of these previous findings supported the concept that a high-dose serum IL-34 level is a risk factor for both RA and OA. Thus, IL-34 has the classical actions, including a possibility to generate bone erosion, and may play a key role in the formation of RA and OA. However, the role of low-dose IL-34 in bone metabolism was still unclear. In our study, we focused on the relationship between low-dose IL-34 and bone metabolism, revealing that IL-34 from 0.0001 to 0.01 ng/ml contributed to osteoblastogenesis. The effects of IL-34 on hBMSCs during osteogenesis were evaluated by qRT-PCR and Western blotting analysis, revealing that IL-34 increased osteo-specific genes and proteins at lower concentrations, especially 0.001 ng/ml. ALP staining and ARS are early and late markers, respectively, of osteoblastic differentiation [3, 31, 36]. We found that IL-34 intensified ALP activity and deepened mineralization at lower concentrations, especially at 0.001 ng/ml. Those results suggested that low-dose IL-34 promoted the osteogenesis of hBMSCs in vitro. Meanwhile, we also observed that low-dose IL-34 has no effect on osteoclastogenesis both in vivo and in vitro (Fig. 7).

The specific tyrosine residues were increasingly dimerized and autophosphorylated intracellularly by the association of IL-34 with the extracellular domain of CSF-1R, leading to the accomplishment of kinds of kinases and adaptor proteins. Such players can be found in signaling pathways, including ERK and AKT [37]. These pathways enhance the pleiotropy of IL-34-mediated CSF-1R when cells differentiated, attached, migrated, and proliferated. Furthermore, they stimulate cellular cytoskeletal organization and survival and subsequently modulate the specific genes expression [38]. As shown in Fig. 2, our research described an obvious increase in the expressions of P-AKT and P-ERK during the hBMSC-driven differentiation with endogenous IL-34.

Various specific molecules could activate PIK/AKT pathways for MSCs, such as IL-37, PDGFRβ, and NANOG [3, 39, 40]. Several studies have emphasized the key role of the PI3K/AKT signaling pathway for all of the periods of osteogenic differentiation, maturation, and bone formation [37, 38, 41]. Not only chondrocyte differentiation would be impaired, but also longitudinal bone growth would be inhibited by blocking the PI3K/AKT signaling pathway [3, 42]. With the activation of the PI3K/AKT signaling pathway, IL-34 switched the phenotype of Kupffer cells from M1 to M2 in vitro [43]. Chen et al. mentioned that IL-34, which is expressed and secreted by embryonic stem cells, may be responsible for ESC-promoted macrophage survival by activating the ERK1/2 and PI3K/AKT pathways [44]. In this study, we found that IL-34 enhanced bone formation by activating the PI3K/AKT signaling pathway. An inhibitor specific for the PI3K/AKT pathway significantly inhibited P-AKT. Western blotting analyses, ALP staining, ARS, and IF analyses further confirmed the regulatory role of the IL-34-PI3K/AKT axis in the osteogenic differentiation of BMSCs.

The ERK pathway, one of MAPK signaling pathways, is an important signal transducer in the regulation of the osteoblastogenesis of MSCs and bone metabolism [45]. It has been reported that major secreted ligands that regulate osteoblast activity seem to serve partly via the ERK pathway [46]. In accordance with previous studies, plenty of specific molecules, such as PDGFRβ, FOXA2, and Withanolide B, could activate ERK pathways for MSCs [39, 47, 48]. The expression levels of RUNX2 and Osterix are firmly associated with ERK phosphorylation [49]. Matsushita et al. found a critical role for ERK in osteoblast mineralization because mice with *Erk1* and *Erk2* deletions display dramatically reduced bone mineralization [50]. Further, IL-34 modulates rheumatoid synovial fibroblast proliferation and migration via the ERK/AKT signaling pathway [51]. According to the experimental results, such as western blotting, ALP staining, ARS, and IF analyses, we found ERK signaling pathway is firmly correlated to osteogenesis in hBMSCs. In order to further verify our results, an inhibitor (U0126) specific for ERK signaling pathway was applied. Treatment with U0126 blocked ERK1/2 phosphorylation and significantly decreased RUNX2, COL1A1, P-ERK, ALP activity, and mineralized nodule formation when compared with the control group. Based on the results above, we demonstrated that ERK signaling pathway is quiet important in IL34-induced osteogenesis in hBMSCs.

The relationship between cytokines and bone metabolism has been demonstrated by several studies [13, 21, 23, 35]. Nevertheless, this is the first study, to the best of our knowledge, to demonstrate the effect of low-dose IL-34 on the dynamic balance of bone metabolism, as shown in Fig. 6h, and we believe it will contribute to the understanding of the relationship between bone fraction and inflammation and between the MSCs and inflammation. Furthermore, our findings provide a new thinking and experiment data for clinical studies in the treatment of many aspects of bone healing, namely, a sustained release system of low-dose IL-34. Unfortunately, we did not investigate the impact of IL-34 on signaling molecules, such as IL-1, IL-6, IL-8, and TNF-a, that shape the inflammatory microenvironment. Moreover, the mechanisms of crosstalk between PI3K/AKT and ERK/MAPK signaling are not fully clarified and require further investigation in future studies. Finally, a rat OVX model used in vivo and mBMMs used for in vitro experiments may reveal the effect of low-dose IL-34 on osteoclastogenesis. Even though the percentage identity of human IL-34 with the rat and mouse IL-34 are 72% and 71%, respectively [12], there are still biological structural difference among different species. Thus, further studies are needed.

## Conclusions

Collectively, our study first demonstrate that low-dose IL-34 regulates osteogenesis of hBMSCs partly via the PIK/AKT and ERK signaling pathway and enhances fracture healing, with neither promoting nor preventing osteoclastogenesis in vitro and osteoporosis in vivo.

## Supplementary Information

**Additional file 1: Figure S1.** A CCK-8 was used to examine the vability after mBMMs cultured with low-dose IL-34 for 1 and 5 d. All of the experiments were independently accomplished no less than three times. Data are means ± SD. $*P* < 0.05 vs. the 1 d group at the same concentration. **Figure S2.** Cells cultured with 30 ng/ml IL-34 or M-CSF in complete α-MEM (100 ng/ml RANKL). (A-C) TRAP staining demonstrated that IL-34 was working in our experiments. **Figure S3.** To confirm the ratio of transduction, expression of GFP was determined by Immunofluorescence was performed.

## Data Availability

The datasets used and/or analyzed during the current study are available from the corresponding author on reasonable request.

## Abbreviations

MSCs: Mesenchymal stem cells
BMSCs: Bone marrow-derived mesenchymal stem cells
hBMSCs: Human bone marrow-derived mesenchymal stem cells
BMMs: Bone marrow-derived macrophages
mBMMs: Mice bone marrow-derived macrophages
CCK-8: Cell counting kit-8
ALP: Alkaline phosphatase
qRT-PCR: Quantitative RT-PCR
ARS: Alizarin red staining
OVX: Ovariectomized
IL-6: Interleukin-6
IL-8: Interleukin-8
IL-34: Interleukin-34
CSF-1R: Colony-stimulating factor-1 receptor
RA: Rheumatoid arthritis
OA: Osteoarthritis
IA: Inflammatory arthritis
RANKL: Receptor activator of NF-κB ligand
α-MEM: Minimum essential medium Eagle Alpha modification
M-CSF: Macrophage colony-stimulating factor
rhIL-34: Recombinant human IL-34
COL1A1: Collagen type I a 1 chain
GAPDH: Glyceraldehyde-3-phosphate dehydrogenase
RUNX2: Runt-related transcription factor 2
ERK1/2: Extracellular signal-regulated kinase 1/2
OCN: Osteocalcin
OPN: Osteopontin
AKT: Protein kinase B
NFATc1: Nuclear factor of activated T cells cytoplasmic 1
PGC1β: Peroxisome proliferative actives receptor γ gamma coactivator 1β
ODM: Osteogenic differentiation medium
PBS: Phosphate buffer saline
ddH_2_O: Double-distilled water
TRAP: Tartrate-resistant acid phosphatase
OE: Overpress
NC: Negative control
ELISA: Enzyme-linked immunosorbent assay
BV/TV: Trabecular bone volume per total volume
BS/BV: Trabecular bone surface per bone volume
Tb.Th: Mean trabecular thickness
Tb.N: Mean trabecular number
Tb.Sp: Mean trabecular separation
Conn-Dens: Mean connectivity density
SP7/Osterix: Zinc finger transcription factor
