# Supplementary material for: Low-dose IL-34 has no effect on osteoclastogenesis but promotes osteogenesis of hBMSCs partly via activation of the PI3K/AKT and ERK signaling pathways
Source: Stem Cell Res Ther. 2021 May 4;12:268. doi: 10.1186/s13287-021-02263-3 (PMC8097863; doi:10.1186/s13287-021-02263-3)
Supplement: Supplementary file 1 — Additional file 1: Figure S1. A CCK-8 was used to examine the vability after mBMMs cultured with low-dose IL-34 for 1 and 5 d. All of the experiments were independently accomplished no less than three times. Data are means ± SD. $P < 0.05 vs. the 1 d group at the same concentration. Figure S2. Cells cultured with 30 ng/ml IL-34 or M-CSF in complete α-MEM (100 ng/ml RANKL). (A-C) TRAP staining demonstrated that IL-34 was working in our experiments. Figure S3. To confirm the ratio of transduction, expression of GFP was determined by Immunofluorescence was performed. [file 13287_2021_2263_MOESM1_ESM.docx]

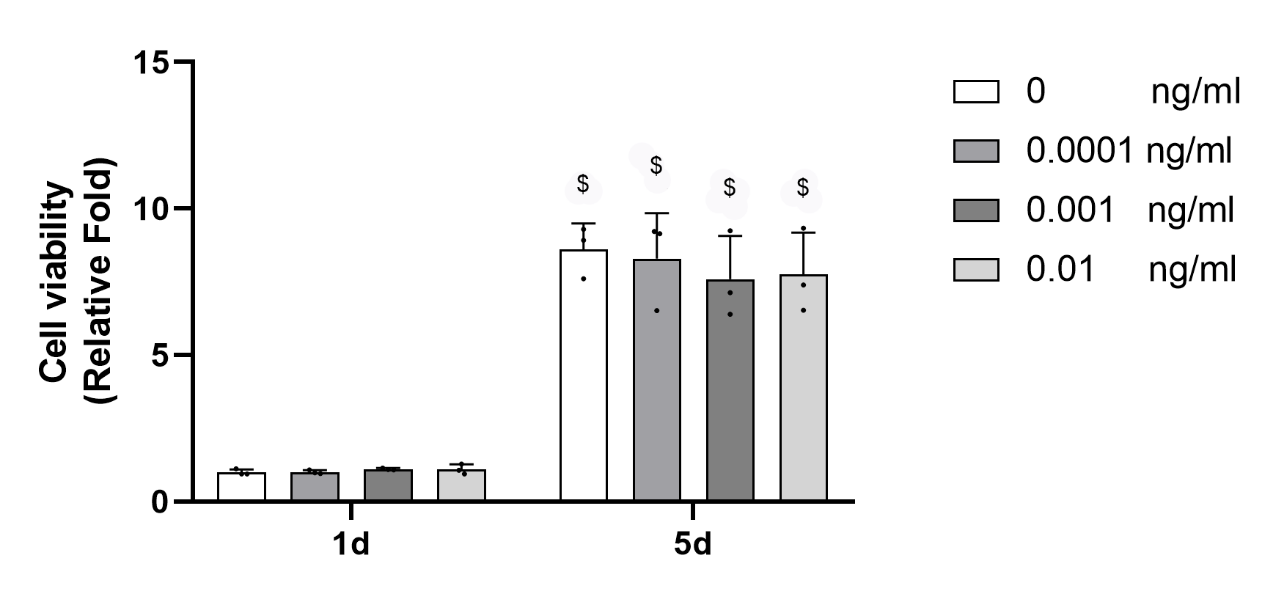


**Supplemental Figure 1**. A CCK-8 was used to examine the vability after mBMMs cultured with low-dose IL-34 for 1 and 5 d. All of the experiments were independently accomplished no less than three times. Data are means ± SD. ^$^P < 0.05 vs. the 1 d group at the same concentration.


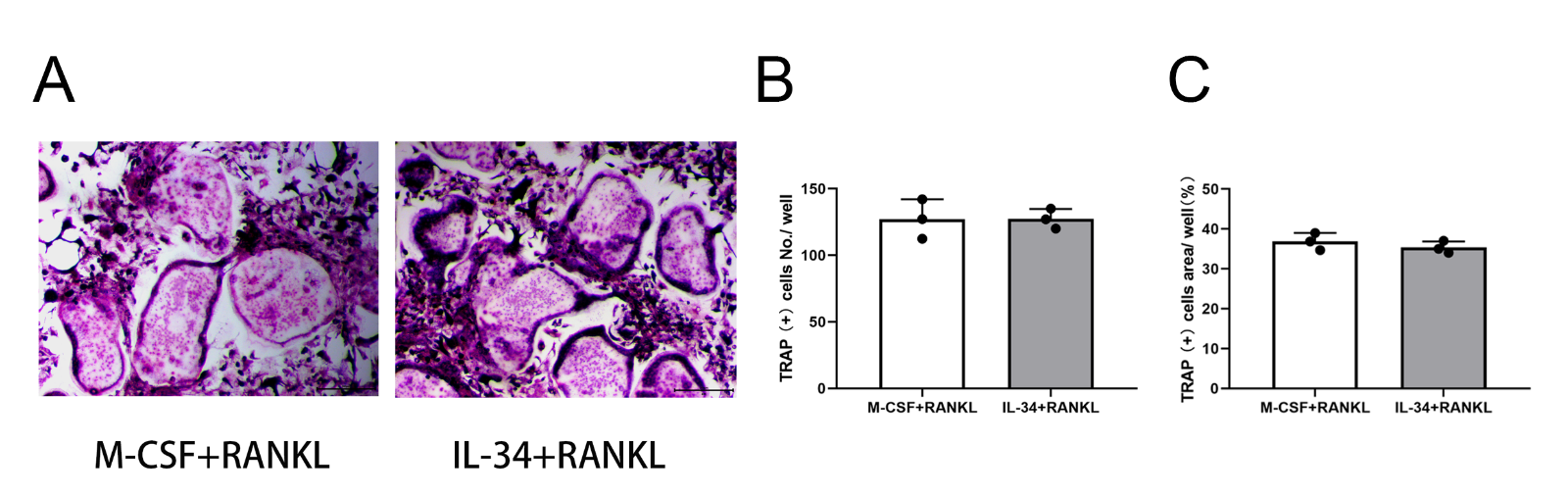


**Supplemental Figure 2**. Cells cultured with 30 ng/ml IL-34 or M-CSF in complete α-MEM (100 ng/ml RANKL). (**A-C**) TRAP staining demonstrated that IL-34 was working in our experiments.


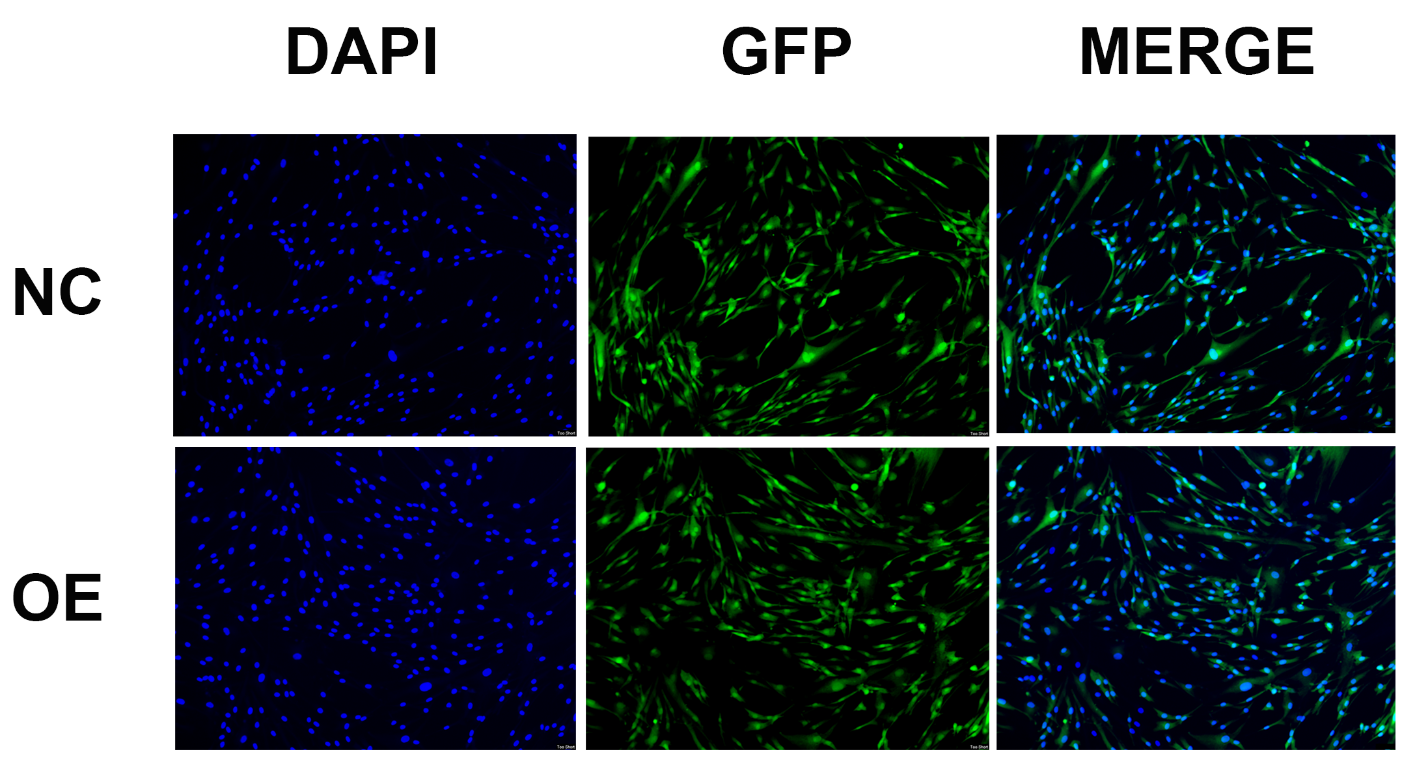


**Supplemental Figure 3**. To confirm the ratio of transduction, expression of GFP was determined by Immunofluorescence was performed.
